# Virus-like Particles as Vaccines for Allergen-Specific Therapy: An Overview of Current Developments

**DOI:** 10.3390/ijms25137429

**Published:** 2024-07-06

**Authors:** Helena Berreiros-Hortala, Gonzalo Vilchez-Pinto, Araceli Diaz-Perales, Maria Garrido-Arandia, Jaime Tome-Amat

**Affiliations:** 1 Centro de Biotecnología y Genómica de Plantas, Universidad Politécnica de Madrid (UPM)-Instituto Nacional de Investigación y Tecnología Agraria y Alimentaria (INIA/CSIC), Campus de Montegancedo UPM, Pozuelo de Alarcón, 28223 Madrid, Spain; h.berreirosh@upm.es (H.B.-H.); gonzalo.vilchez@upm.es (G.V.-P.); araceli.diaz@upm.es (A.D.-P.); maria.garrido@upm.es (M.G.-A.); 2 Departamento de Biotecnología-Biología Vegetal, Escuela Técnica Superior de Ingeniería Agronómica, Alimentaria y de Biosistemas, UPM, 28040 Madrid, Spain

**Keywords:** virus-like particles, therapy, allergy

## Abstract

Immune engineering and modulation are the basis of a novel but powerful tool to treat immune diseases using virus-like particles (VLPs). VLPs are formed by the viral capsid without genetic material making them non-infective. However, they offer a wide variety of possibilities as antigen-presenting platforms, resulting in high immunogenicity and high efficacy in immune modulation, with low allergenicity. Both animal and plant viruses are being studied for use in the treatment of food allergies. These formulations are combined with adjuvants, T-stimulatory epitopes, TLR ligands, and other immune modulators to modulate or enhance the immune response toward the presented allergen. Here, the authors present an overview of VLP production systems, their immune modulation capabilities, and the applicability of actual VLP-based formulations targeting allergic diseases.

## 1. Introduction

Allergy is a worldspread disease characterized by an exacerbated immune response to a normally tolerated molecule. The prevalence of allergies has increased in the last years, affecting approximately 5% of the global population, with higher concern when considering infants [1,2]. In this context, the allergens responsible for eliciting allergic reactions in susceptible individuals are predominantly food molecules, consisting mainly of proteins and/or lipoproteins [3,4,5]. Although medical treatments for food allergies have made considerable progress [1,6], healthcare professionals universally recommend allergen avoidance as a primary measure [7].

In recent years, there has been a growing emphasis on taking advantage of nanotechnology for allergen detection, diagnosis, and treatment using allergen-specific immunotherapy (AIT) [8,9]. AIT involves gradually administering small amounts of the allergen to the patient, with the goal of training the immune system to tolerate its presence. While AIT remains the only treatment capable of eradicating certain allergic phenotypes, its application in food-allergic patients entails significant challenges, such as the need to achieve better benefit-to-risk ratios before widespread adoption in routine clinical practice [10,11,12].

Hence, novel approaches have been proposed to enhance the effectiveness, safety, and convenience of allergy therapy, such us (a) exploring new administration routes; (b) utilizing allergens, hypoallergens, allergoids, and peptide-based vaccines produced via recombinant methods; and introducing new adjuvants derived from bacteria and viruses, such as (c) bacterial extracts and (d) TLR ligands and, (e) advancements in formulations and delivery systems, such as virus-like particles (VLPs), DNA vaccines, aggregates, nanoparticles, or liposomes [1,13].

In the field of nanobiotechnology, virus-like particles have arisen as a powerful tool for a variety of applications [14,15,16]. VLPs consist of the virus capsid protein but without the corresponding encapsidated nucleic acid [17]. They serve as efficient delivery platforms, capable of transporting a wide variety of cargos due to their controlled self-assembly and adaptable architectures [16,18,19,20]. The nature of VLPs makes them generally highly biocompatible and biodegradable, which is a great advantage compared to other nanoparticles with intended use in therapy [21].

Cargos of interest can be attached to VLPs via genetic engineering or chemical fusion [22], enhancing concentration and efficacy while minimizing the need for potentially harmful adjuvants and the presence of the side effects [23,24]. The presence of these nanostructures with highly repeated domains is known to effectively activate the immune system, akin to vaccines, stimulating both B-cell and dendritic cell responses [25,26]. Regarding toxicity, VLPs are generally considered safe, but careful evaluation is needed [21]. The use of VLPs as a vaccine as promising therapeutic tools is already in trial for diseases such as Alzheimer’s [27,28], arthritis [29], atherosclerosis [30,31], cancer [32,33,34], and, since their early days, for infectious diseases such as malaria, COVID, or papillomavirus [35].

**Figure 1:**
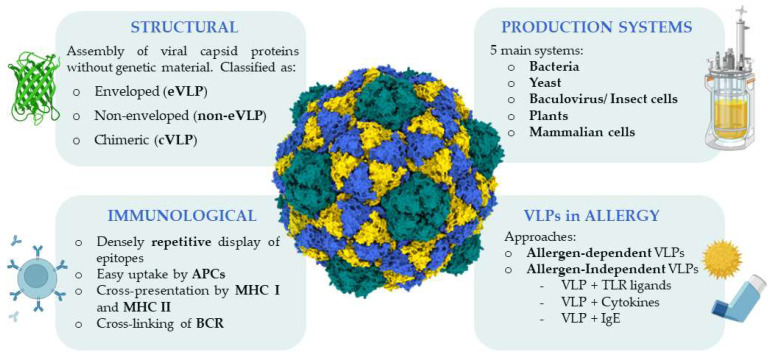
Summary of the main issues reported throughout this review: Structural classification, different production systems, the immune mechanisms associated, and the application of VLPs in the treatment of allergies. Created with BioRender.com and ChimeraX v1.17.

This manuscript aims to review and update the use of these powerful tools in the treatment of allergic diseases. To this end, the review presents a brief overview of what VLPs are, including their structure and function, the systems used to produce them, and the current state of the art in VLPs and allergy research (Figure 1).

## 2. VLPs as Vaccine: Success Stories

### 2.1. Structural Features

**Figure 2:**
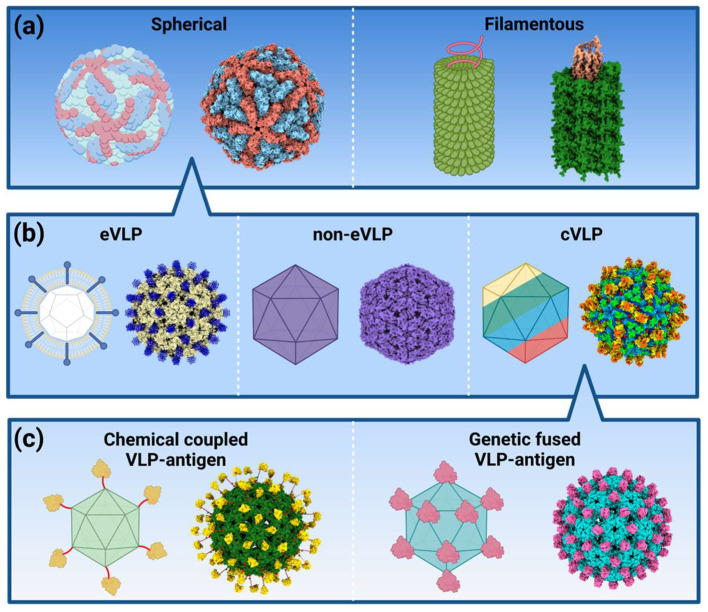
Schematic and modeled structural classification of VLPs. (**a**) According to their morphology, VLPs can be spherical or filamentous. (**b**) Both spherical and filamentous VLPs can be distinguished according to the structure of the assembled particle: eVLP (membrane in wheat color, glycoproteins in blue), non-eVLP (capsid in purple), or cVLP (capsid in rainbow). (**c**) cVLPs can be further classified according to the type of conjugation, such as chemically coupled (capsid in green, linker in red, epitope in yellow) or genetically fused (capsid in light blue, epitope in pink). Created with BioRender.com and ChimeraX v1.17.

As mentioned above, VLPs are viral capsids without genetic material, preventing their replication and, therefore, infection capacity. These structures are formed by viral capsid proteins or other self-assembly proteins [17,36,37]. VLPs can be both spherical and filamentous structures. Based on the structure of the coated protein, both types of VLPs can be classified as enveloped (eVLP), non-enveloped or naked (non-eVLP), and chimeric (cVLP) [38] (Figure 2).

eVLPs are complex particles that require the host membrane for envelopment. Thus, in addition to a protein capsid, they will be coated with a lipid bilayer. In some cases, the correct assembly of the eVLP involves specific glycoproteins [24,39]. eVLPs are stable and flexible structures, making them ideal for transporting components; they can be attached to the outermost layer or carried inside. However, the production of these particles is highly complex and will depend on the production system [40].

Non-eVLPs are membraneless particles that arise from the self-assembly of one or more proteins. While there are simple particles composed only of the main nucleocapsid protein, there are complex multiprotein particles that, in addition to the capsid protein, have auxiliary proteins associated with them [24,41]. In general, these particles have higher stability than eVLPs, as they are less susceptible to environmental changes such as temperature, shear strength, and chemical treatments [40].

Finally, cVLPs result from the assembly of viral components from at least two different viral serotypes, or are the product of recombinant DNA [38]. Spherical cVLPs are becoming highly relevant for the encapsulation and display of molecules. Filamentous cVLPs are also being used due to their high functionalization power, as they are structures composed of thousands of subunits (as opposed to spherical ones, composed of hundreds of subunits) [42]. This approach shows great advantages, as the particle surface is highly modifiable by chemical or genetic approaches [43].

To conjugate an epitope by chemical techniques, a linker that binds at one end to the lysines of the viral capsid is used. The other free end of the linker will bind to the epitope of interest, forming the VLP–epitope complex [44]. However, the highest yielding approach consists of gene fusion methods. For this, the VLP is expressed with the sequence of the desired epitope incorporated into the sequence of the viral proteins themselves [17].

The major disadvantage of both conjugation methods is related to the addition of an exogenous component. Due to this, the particle may cause misfolding of viral proteins or decrease their ability to self-assemble [45]. On one hand, chemical conjugation facilitates an easy broad spectrum of diverse formulas, based on the production of a single common batch with controlled conditions [46]. Therefore, the same platform can be used for many objectives. This is a very interesting point when facing a large scale of production and the possibilities of treating different diseases. Moreover, chemical conjugation will better preserve the stability and architecture of VLP compared to genetic coupling [47]. On the other hand, genetic conjugation offers a controlled molecular ratio of the VLP–epitope complex, the possibility of disassembling/reassembling the formula, and more safety in terms of the presence of free antigen. In addition, this methodology dispenses with pH-dependent chemical reactions and the use of toxic reagents [48].

### 2.2. Challenges in the Production of VLPs

**Scheme 1:**
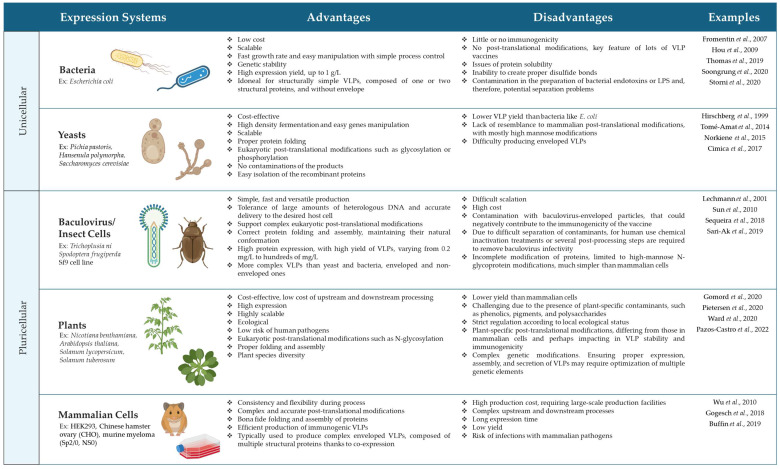
Advantages and disadvantages of different expression systems for the recombinant production of VLPs. Icons created with BioRender.com [12,32,60,61,62,63,64,65,66,67,68,69,70,71,72,73,74,75,76,77].

VLPs can be obtained from the parental virus by removing the genetic material, although this approximation may show several safety issues. The majority of VLPs produced are based on heterologous systems by recombinant production of the capsid protein with or without helper proteins [38]. Recombinant production of VLPs can be obtained from, mainly, five systems: (a) bacteria, (b) yeast, (c) baculovirus/insect cells, (d) mammalian cells, and (e) plants, each with distinct advantages and challenges. The key determinants for choosing a suitable synthesis system to produce VLPs are the yield and the ability to scale up, the requirement on the structural complexity and immunogenicity of VLPs, process stability, flexibility for modification, safety, and cost [49,50]. The advantages and disadvantages of each system are summarized in Scheme 1.

Briefly, bacterial and yeast cells are commonly chosen for their high production yields, but limitations in achieving complex post-translational modifications (PTMs) and their reduced immunogenicity hinder their widespread adoption [51]. In contrast, baculovirus/insect cell (B/IC) systems offer versatility and high expression levels, but concerns remain regarding the co-production of baculovirus-enveloped particles and downstream processing complexities [52]. Mammalian cells enable complex PTMs, making them advantageous for VLP production, although their higher production costs and lower controllability pose challenges [52,53]. Transgenic plants offer benefits such as low processing costs and increased safety but face limitations in PTMs and expression levels. Additionally, methods like transfection and transduction play critical roles in the production, each with unique considerations for achieving high protein yields [54,55].

Another alternative system to produce VLPs can be the cell-free system. This has become an option with great potential industrial importance, especially for producing proteins that are toxic or for being able to introduce modifications with non-biological amino acids [51].

Besides the previously mentioned factors for selecting a system for VLP production, safety and the absence of side effects are particularly important considerations. These concerns involve both the production system and the nature of the VLP being produced [56,57]. For instance, eVLPs, due to their enveloped nature, may contain impurities that could cause undesired side effects. While non-eVLPs and cVLPs are also susceptible to impurities (such as those within the inner cavity or from non-specific binding), these impurities are easier to remove during the purification process [58,59]. High-scale VLP production following cGMP practices is tightly regulated by the Food and Drug Administration (FDA) and the European Medicines Agency (EMA) of the Guidance for Industry of the Viral Safety Evaluation of Biotechnology Products from Cell Lines of Human or Animal Origin Q5A2(R2).

Finally, live animals can be used as another strategy for the expression of VLP vaccines, with more complete post-translational modifications. This system has a relatively low cost, and the strategy offers great robustness and easy mass production compared to other systems. For example, the expression system of the protozoan *Leishmania tarentolae* offers post-translational modifications similar to those of mammals and is not pathogenic for humans [78].

### 2.3. Immunological Mechanisms of VLPs

VLPs can act as antigens just like the viruses from which they originate. This can be favorable when you want to enhance the immune response (immunotherapy against tumors), but it can be harmful when trying to improve the symptoms of an autoimmune disease [38].

The size (between 20 and 200 nm) and shape of VLPs greatly facilitate a wide variety of interactions (ionic, hydrophobic, and hydrophilic) with the surface of the antigen-presenting cells (APCs), favoring their absorption [21]. Their particulate structure and repetitive antigens allow them to be absorbed efficiently and can be presented in both MHC class I and class II molecules [26,53]. The repetitive arrangement of the surface of VLPs also allows them to be recognized by B-cell receptors (BCRs), being able to induce a strong humoral response [79]. Therefore, VLP-derived peptides presented by MHC class II result in the activation of CD4+ T helper cells and in the generation of protective IgG antibody titers. Interestingly, several studies demonstrated that VLPs can also induce efficient cytotoxic T-cell responses by cross-presentation [26,80,81,82].

Furthermore, the repetitive epitopes that VLPs present can also be recognized by the innate immune system, recruiting humoral components such as natural IgM and the complement system [83]. Activation of this humoral response can also enhance B-cell activation and promote B-cell-mediated antigen deposition in follicular dendritic cells (FDC), which is essential for the formation of germinal centers and, therefore, for the generation of long-lived memory and plasma B cells. Some immunostimulatory agents or other adjuvants can be packaged with VLPs to enhance the response even more [84]. Therefore, VLPs may encapsulate drugs within their structures or attach them to the surface, offering controlled release and specific targeting of cells or other tissues by the incorporation of specific ligands [85]. In comparison, other nanovehicles may offer similar drug delivery capabilities, but with differences in release kinetics, targeting abilities, and loading capacities based on the type. These vehicles can also be functionalized to improve targeting [86].

Hence, VLPs emerge as an attractive option for vaccination, compared to live or attenuated viruses. The spectrum of production systems, the time for production (3–12 weeks), and their immunogenic properties make them a promising platform for vaccination [44,53]. Owing to these facts, the FDA approved the first VLP-derived vaccine, against Hepatitis B [87,88]. Since then, other VLP-based therapies have reached the market: Recombivax HB^®^ (Merck & Co., Inc., Rahway, NJ, USA) [89], Engerix^®^ B (GlaxoSmithKline, Brentford, UK) [90], and Sci-B-Vac^TM^ (VBI Vaccines Inc., Cambridge, Massachusetts, USA) [91] for hepatitis B; Gardasil^®^ (Merck & Co., Inc., Rahway, NJ, USA) [92], Gardasil9^®^ (Merck Sharp & Dohme LLC, Rahway, NJ, USA) [93], and Cervarix^®^ (GlaxoSmithKline, Brentford, UK) [94] for human papillomavirus (HPV); Hecolin^®^ (Xiamen Innovax Biotech, Haicang, Xiamen, China) [95] for hepatitis E; and, recently, Mosquirix™ (GlaxoSmithKline, Brentford, UK) [96] for malaria.

## 3. Application of VLPs in the Treatment of Allergic Diseases

The final goal of the use of VLPs in the treatment of allergies is to potentially induce immune tolerance and desensitization, promoting regulatory T-cell responses and reeducating the immune system towards a more tolerogenic state. To reach this objective, there are several approaches using modified VLPs, displaying allergenic proteins, peptides, and other molecules on their surface, by genetical fusion or chemical conjugation [37].

These formulas can also play a dual role in allergy vaccination; first, acting as an adjuvant to facilitate antigen presentation; second, helping to reduce the Th2 response, skewing the immune system towards a Th1 type response, which is important for sustained immunity to the allergen [84]. The repetitive display optimizes BCR cross-linking stimulation to produce IgG with high affinity, which is required for successful allergen neutralization [97]. In addition to that, thanks to their inherent adjuvant properties, they enhance the immune response and promote stronger and longer-lasting immunomodulatory effects [98].

VLP-based allergy treatments are reported to trigger fewer side effects compared to traditional allergen immunotherapy, with reduced systemic reactions. Allergens that have been coupled to VLPs seem to be unable to provoke anaphylactic reactions in allergic individuals because of the physicochemical differences between free allergens and the VLP-coupled ones [84]. It has also been demonstrated in vitro that allergens bound to VLPs are unable to activate mast cells, showing a strong ability to bind to surface-linked IgE. This may indicate that repetitively displaying allergens on VLPs increases their immunogenicity while reducing their potential to cause anaphylactic reactions by the inhibition of the IgE-mediated activation of mast cells [99].

There are two different main approaches for the allergy treatment with VLPs: allergen-dependent VLPs and allergen-independent VLPs (Table 1).

### 3.1. Allergen-Dependent VLPs

This classification comprises all VLPs that induce an allergen-dependent immunomodulation. It is based on the direct binding of VLP vehicles with specific full-length allergens or B-cell epitopes. The aim of this formula is to induce allergen-specific T-cell tolerance and the production of blocking antibodies [100]. Allergens can be displayed on the surface, but they can also packed into VLPs [37]. However, the latter is a less frequently used strategy.

This approach shows many advantages, such as the precise targeting of the molecular cause of the disease, without affecting unrelated immune responses [100]. In some murine models presented in Table 1, the prevention of systemic anaphylaxis was observed in mice treated with these platforms [97,100,101,102]. Related to adverse effects, allergens displayed on the surface of VLPs induce a weaker degranulation in effector cells compared to soluble allergens at equivalent concentrations [99]. However, it is extremely important to ensure the stability of the particle because an undesired disassembly could release a full-length allergen, which may lead to effector cell activation and anaphylaxis [103]. Moreover, the use of epitopes of major allergens is also an interesting alternative to reduce the chances of inducing unwanted reactions [97].

Several studies have been carried out about the use of these particles as allergy immunotherapy. For instance, VLP coupled to the peach major allergen Pru p 3 was produced as an immunotherapeutic formulation against peach allergy [12]. The formula consisted of the coat protein of the turnip mosaic virus (TuMV) and Pru p 3, separated by a linker. It was introduced into a highly expressed transient vector, followed by agroinfiltration in *Nicotiana benthamiana*. Sublingual administration of the formula in allergic mice effectively reduced some serological markers associated with allergic responses, such as anti-Pru p 3 serum IgE and serum IgG2a, with no toxicity associated. The resulting formulation exerts remarkable immunomodulatory properties without the need for potentially hazardous adjuvants [23].

Relating to peanut allergy, VLPs derived from the cucumber mosaic virus (CuMV) were used to display the peanut allergen Ara h 1, Ara h 2, or Ara R, the extract of roasted peanut [64]. The allergens were chemically coupled to the viral platform. Peanut-allergic mice were vaccinated subcutaneously with each formula and the three VLPs led to the prevention of an anaphylactic outcome. This treatment induced the development of protective IgG responses in allergic mice. The proposed mechanism is based on the competition between IgG and IgE for the allergen.

**Table 1:** Recent advances in the development of allergy immunotherapies based on the use of VLPs.

ALLERGEN-DEPENDENT				
VLP	Target	Organism	Observed Effects	Reference
**Vaccine BM32** PreS domain of Hepatitis B Virus (HBV)	Phl p 1, 2, 5, 6	Human phase II clinical trial	Increase in IgG4 allergen-specific antibodies No IgE levels enhanced	ClinicalTrials.gov Identifier: NCT01538979
Monoley-murine-Leukemia Virus (MLV)	Art v 1	Mouse	Surface exposed Art v 1 VLP induces allergen-specific antibodies Induction of Th1/Treg response	[103]
Monoley-murine-Leukemia Virus (MLV), displaying GM-CSF	Ova-derived peptides	Mouse	Expansion of CD11b+ cells within bone marrow Induction of antigen-specific CD4+ and CD8+ T-cell proliferation	[75]
Acinetobacter phage AP205 fused to SpyCatcher (SpyCatcher-VLP)	Der p 2	Mouse	Blocking of allergen-specific IgG Prevention of specific IgE	[61]
**HypoCat™** Cucumber Mosaic Virus engineered with tetanus toxoid universal T-cell epitope (CuMV_TT_)	Fel d 1	Cat	Development of neutralizing antibodies against Fel d 1 Sustained specific IgG antibody response Reduction in symptoms in cat owners	[60,104]
Cucumber Mosaic Virus engineered with tetanus toxoid universal T-cell epitope (CuMV_TT_)	Ara h 1, 2, R	Mouse	Protection of peanut-sensitized mice against anaphylaxis to the whole peanut extract Induction of specific IgG antibodies	[64]
TM/CT domain of Influenza Virus hemagglutinin	Der p 2	Mouse	Strong IgG response Low basophil degranulation of human sera	[72]
Hepatitis B core antigen (HBcAg)	Che a 3-derived peptide	Mouse	Lack of IgE-binding and basophil degranulation activity Induction of rChe a 3-related IgG antibody Low polcalcin-specific IgE	[105]
Turnip Mosaic Virus (TuMV)	Pru p 3	Mouse	No adjuvants needed Reduction in allergen-specific IgE and IgG2a	[12]
Cucumber Mosaic Virus engineered with tetanus toxoid universal T-cell epitope (CuMV_TT_)	Ara h 2	Mouse	Significant anti-Ara h 2 IgG response Confer systemic protection	[102]
PreS domain of Hepatitis B Virus (HBV)	Bet v 1/Mal d 1-derived peptides	Rabbit	Lack of IgE reactivity and allergenic activity Presence of neutralizing antibodies to both allergens at the same time	[106]
Cucumber Mosaic Virus engineered with tetanus toxoid universal T-cell epitope (CuMV_TT_)	Ara h 2	Human phase I clinical trial	Recruiting candidates	ClinicalTrials.gov Identifier: NCT05476497
**ALLERGEN INDEPENDENT: Immunomodulation by TLR ligands**				
Bacteriophage Qβ-derived VLP	CpG-motif G10 (TLR9 ligand)	Human, phase IIb clinical trial	Improvement of rhinoconjunctivitis symptoms in dust mite-allergic patients	ClinicalTrials.gov Identifier: NCT00800332
Bacteriophage Qβ-derived VLP	CpG-motif G10 (TLR9 ligand)	Human, phase II clinical trial	Improvement of asthma symptoms and relief medications in allergic patients	ClinicalTrials.gov Identifier: NCT00890734
**ALLERGEN INDEPENDENT: Neutralize cytokines**				
Hepatitis B core antigen (HBcAg)	Recombinant IL-13 peptide	Mouse	Partial suppression of induced airway remodeling features Production of anti-IL13 antibodies	[107]
Cucumber Mosaic Virus engineered with tetanus toxoid universal T-cell epitope (CuMV_TT_)	Recombinant IL-5	Horse	Induction of neutralizing anti-IL-5 IgG Reduction in eosinophil inflammation in lesions Response maintained over a year	[108,109,110]
**ALLERGEN INDEPENDENT: Neutralize allergen-specific IgE**				
Bacteriophage Qβ-derived VLP	IgE peptides Y and P	Mouse	Strong antibody response to IgE peptides by TLR7 activation Production of blocking anti-IgE antibodies	[19]
Cucumber Mosaic Virus engineered with tetanus toxoid universal T-cell epitope (CuMV_TT_)	Synthetic mouse IgE-Fc fragments	Mouse	High amount of anti-IgE antibodies Less IgE bound to FcεRI on the surface of basophils	[111]

These formulas could prevent the crosslinking of IgE with the FcεRI receptor, as well as inducing the direct binding of IgG with the inhibitory receptor FcγRIIb, present on mast cells and basophils. In the presence of high levels of anti-allergen IgG antibodies, IgG–immune complexes form and bind FcγRIIb, causing the inhibition of IgE-mediated signals. This emphasizes the role of allergen-complexed IgG in the regulation of anaphylaxis. Moreover, this approach induced protection against the whole peanut extract, made up of multiple allergens, not only against the allergen displayed by the VLP itself [64].

Another strategy to treat peanut allergy was the genetic fusion of the allergen Ara h 2 cDNA to CuMV VLP subunits [102]. This is different from the previously described formula, where the coupling was carried out by chemical reactions. Subcutaneous immunizations in peanut-sensitized mice with this formula resulted in an anti-Ara h 2 IgG response and the protection against both systemic and local anaphylaxis. This reconfirmed the crucial role of the inhibitory FcγRIIb receptor in cross-protection against peanut allergens other than Ara h 2, by inhibiting FcγRIIb function and observing a loss of protection. The serum from treated mice could inhibit the binding of high affinity anti-Ara h 2 IgE. In addition, the authors stated that their formula can protect against systemic anaphylaxis when used in a prophylactic immunization regimen. This hypothesis was tested by the application of the vaccine to naïve mice, who were not previously allergic to peanut extract. After treatment, the mice were sensitized, and the challenge was performed weeks later. The prophylactic immunization conferred protection against anaphylaxis, maintaining protective titers of IgG antibodies against peanut. This formula has now entered clinical development under the name of the PROTECT clinical trial [112] (ClinicalTrials.gov Identifier: NCT05476497).

Regarding to grass pollen allergy, one of the most relevant developed vaccine is BM32 (Biomay AG) [113,114]. The formula includes four recombinant fusion proteins consisting of the hepatitis B virus (HBV)-derived PreS fused to hypoallergenic peptides from the IgE binding sites of the timothy grass pollen allergens Phl p 1, 2, 5, and 6, adsorbed on aluminum hydroxide. A two-year double-blind, placebo-controlled, multicenter immunotherapy clinical trial (phase II clinical trial: ClinicalTrials.gov NCT01538979) was carried out to evaluate the efficacy and safety of the treatment during two consecutive grass pollen seasons. The patients received three subcutaneous injections of BM32 pre-season and a single post-season booster injection in the first year of treatment, to maintain optimal allergen-specific IgG responses. This regimen demonstrated improvement in clinical symptoms of grass pollen allergy. It induced a continuously increasing allergen-specific IgG4 response without activating allergen-specific IgE responses and maintained low stimulation of allergen-specific PBMCs. The IgG4 response was increased in the second year of immunotherapy compared to the first one; thus, clinical efficacy was observed. In addition, allergen-specific pro-inflammatory cytokine responses were not induced [113,114].

Concerning pet allergy, a creative strategy was developed to treat cat allergy in humans by the vaccination of cats with a HypoCat™ vaccine (Saiba Animal Health, Zurich, Switzerland) [104]. The formula is composed of the major cat allergen Fel d 1 and a VLP derived from CuMV with the tetanus toxin-derived universal T-cell epitope tt830-843 (CuMV_TT_) [60]. After subcutaneous vaccination, cats induced a strong specific IgG antibody response in cat owners, leading to the development of neutralizing antibodies against the allergen, thus reducing its endogenous level. The vaccine was well tolerated and had no toxic effects. A persistent reduction in symptoms over the study period was observed in cat owners, and even the total prevention of allergic reactions was reported in some individuals [104].

### 3.2. Allergen-Independent VLPs

These strategies are based on VLPs triggering an allergen-independent immunomodulation. This can be divided into (a) immunomodulation by TLR ligands, (b) VLPs priming the production of neutralizing antibodies against typical allergy cytokines, and (c) VLPs leading to the generation of neutralizing/blocking antibodies against allergen-specific IgE and their receptors, FcεRs [100].

#### 3.2.1. Immunomodulation by TLR Ligands

One of the strategies followed in allergy is trying to switch from a Th2 response and induce a Th1-biased response [115,116]. This can be achieved by exposing VLPs coupled to PRR receptor ligands, such as TLRs. Generally, this strategy is based on the immunological activation of TLR9 [37,100].

This approach is based on the use of VLPs based on single-stranded RNA bacteriophages, which are capable of self-assembly [117]. However, it has been found that the capsid of these viruses can also self-assemble in the presence of synthetic CpG-rich oligodeoxynucleotides (ODNs) that are able to activate TLR9 [118]. In humans, TLR9 is expressed mainly by plasmacytoid dendritic cells (pDCs) and B cells. While pDCs generate type I interferons (particularly IFN-α) in response to TLR9 activation, the primary outcomes of TLR9 signaling are recognized to be the secretion of cytokines and chemokines that support Th1 immune responses. These include substances such as monocyte inflammatory protein-1, IFN-γ, and the promotion of IgG class switching in B cells [119]. Thus, some experiments began to evaluate the potential of using these CpG-VLPs to reprogram Th2 allergic response to Th1-biased responses.

All published information consists of CYT003, a treatment involving QβG10, a CpG-VLP based on bacteriophage Qβ [120]. Supported by promising previous studies [121] (Clinicaltrials.gov Identifier: NCT00652223), a phase IIb clinical trial involving 299 participants was carried out by the subcutaneous injections of QβG10 in house dust mite-allergic patients with rhinoconjunctivitis symptoms. The treatment was shown to be harmless, and it significantly reduced symptoms compared to the placebo group. In addition, these patients reduced their intake of medication associated with allergic symptoms, improving their quality of life, and showed a 10-fold increase in tolerance to the conjunctival provocation dose in the high-dose group [122] (ClinicalTrials.gov Identifier: NCT00800332).

Based on the same approach, a second study was carried out with QβG10 VLP CYT003. Sixty-three allergic asthmatic patients with moderate or high steroid intake were treated with Qβ subcutaneous injections. All patients who received the treatment improved symptomatically in the first 12 weeks, having controlled asthma after that week, reducing the amount of steroid intake [123] (ClinicalTrials.gov Identifier: NCT00890734).

This type of treatment is ideal for individuals without a clear sensitization profile or who are sensitized to complex allergen sources. However, it is not specific for any type of allergen [100]. In fact, its application in allergy did not last much longer. The latest clinical trial conducted in 2014 yielded data contradictory to the trend hitherto observed. A total of 365 patients with moderate-to-severe asthma, who were being treated with inhaled steroids, were treated subcutaneously with CYT003. No significant improvement over the placebo group was observed [124] (ClinicalTrials.gov Identifier: NCT01673672). Since then, no further clinical trials have been conducted with this type of approach, or they were even withdrawn after starting due to lack of results (ClinicalTrials.gov Identifier: NCT02087644).

#### 3.2.2. VLPs Coupled with Cytokines

This type of formula primes the production of neutralizing autoantibodies against typical allergy cytokines, which are necessary to induce and maintain allergic inflammation. This is achieved by the direct coupling of VLPs with type 2 effector cytokines [37,100]. This approach is based on active immunization. The current main and most widespread competitor is passive immunization with monoclonal antibodies, which has become an important treatment option for atopic and allergic diseases [125,126,127].

Our immune system develops tolerance against our proteins, and therefore, the vaccine against cytokines (and IgE) requires overcoming this tolerance. The binding of cytokines to a source of Th cell epitopes, such as VLPs, can drive specific B-cell responses and trigger the induction of IgG autoantibodies against these inflammatory mediators [37]. This is similar to what happens in carbohydrate conjugate vaccines, where B cells recognize the carbohydrate and Th cells recognize the carrier protein to which the carbohydrates are attached [83]. This formula triggers a long-term and polyclonal response against targeted molecules. Thanks to polyclonality, less induction of anti-drug antibodies is observed, which is one of the main drawbacks of monoclonal antibodies. Moreover, the formula requires a more limited number of vaccine doses [100], and it is quite cost-effective. In terms of immunological response, the administration of VLP bound to Th2 cytokines showed a marked reduction in well-known hallmarks of allergic diseases and asthma in animal models. The active induction of anti-cytokines antibodies was also observed in the majority of VLPs formulas [37,100].

Several formulas of VLPs with cytokines such as IL4 [128], IL-5 [129], IL-13 [79], IL-33 [130], etc., have been tested. One of the most remarkable studies involved the genetic fusion of an IL-13 peptide to the hepatitis B core antigen (HBcAg). The subcutaneous administration of this treatment in mice successfully broke tolerance, inducing the production of anti-IL13-specific antibodies. This led to significantly diminished IL-13 concentrations, fewer inflammatory cells in the bronchoalveolar fluid, and a drop in lung mucus production and collagen deposition. As a result, this VLP significantly reduced lung inflammation, remodeling, and hyperresponsiveness, thus limiting asthma exacerbations in the animal model [78].

More recently, some approaches against IL-5, the master regulator of eosinophils, have been developed. For instance, the subcutaneous vaccination with equine IL-5 coupled to a CuMV_TT_, showed promising results in horses suffering from chronic allergic dermatitis caused by insect bites [108]. The aim was to strongly dampen eosinophil recruitment and expansion. It resulted in the induction of anti–eIL-5 antibody titers. This significantly improved the horses’ symptoms related to this chronic relapsing allergic dermatitis. In addition, one year later, they assessed a second follow-up to analyze the potential for long-term therapy [109]. The horses showed even more improvement in the disease in their second vaccination year, confirming that responses could be maintained over the seasons by yearly vaccination against IL-5. Therefore, this may be a long-term solution for the treatment of eosinophil-mediated diseases. Moreover, the authors guaranteed the safety of the formula, ensuring it did not induce auto-reactive IL-5-specific T-cell responses [110]. This was a successful immunotherapeutic approach in horses, and it might shed light on the development of a similar treatment in humans.

When developing this kind of treatment, there are other aspects to take into consideration. As we mentioned before, the breakage of tolerance may lead to adverse effects, such as autoimmune diseases, immunodeficiency, reactivation of latent infections, or even impact tissue remodeling [100]. In addition, once the administration has been performed, their effects are hard to reverse. This is due to the establishment of long-lived plasma cells or vaccine-induced B-cell memory cells, which would be difficult to remove from that moment on [100]. This is clearly different from monoclonal antibodies, whose administration can be stopped, and the effects will disappear after some weeks, without further complications [131]. Another potential issue is the possibility of potentiating the function of cytokines instead of mitigating it, due to an increase in the activity of the cytokines by prolonging their half-life, for example. In addition, some cytokines have been proved to retain their biological activity bound to VLPs, leading to potential adverse effects, such as cytokines storm [100], while other VLPs can be used to treat the cytokine storm syndrome [132,133].

#### 3.2.3. Neutralizing/Blocking Antibodies against Allergen-Specific IgE

The last strategy to counteract allergies is based on the elimination of IgE-producing cells or the neutralization of the function of IgE itself. Tolerance to IgE or FcεRs could be altered by inducing autoantibodies or blocking antibodies [37]. This can be achieved by targeting IgE, its specific receptor FcεRs, and IgE-producing B/plasma cells [100]. Several studies have already used these approaches, with positive results.

A first example is a study in 2007, where three synthetic peptides of the human IgE receptor-binding site of IgE conjugated with HBsAg VLP were produced. This vaccine was injected subcutaneously to rats and mice sensitized to trichosanthin. The vaccinated animals presented low IgE-antibody levels in serum, and they generated blocking FcεR antibodies. However, they did not interact with FcεR-bound rat IgE, showing the non-anaphylactogenicity of the induced autoantibodies [134].

Subsequent studies have shown the importance of innate danger signals in VLP-derived vaccines. For example, it has been demonstrated that QβG10 conjugated with two IgE peptides could stimulate the innate immune system in mice, resulting in the production of blocking anti-IgE antibodies. However, this effect was significantly reduced in TLR7 knockout mice. When mixed with adjuvants such as alum or CpG, no reduction in antibody production was observed in the TLR7 knockout mice [19].

However, few other examples could be found until 2024, when Gharailoo et al. (2024) developed a CuMV_TT_ VLP immunotherapy with chemically bound IgE-Fc fragments. This new strategy was tested in Fel d 1-sensitized mice. Mice immunized with these subcutaneous vaccines produced anti-IgE IgGs and blocked anaphylaxis upon challenge with the allergen [111].

This kind of methodology has the great advantage of generation of blocking antibodies, which inhibit the binding between IgE and its receptors and reduce IgE serum levels. However, no human clinical trials have been conducted. In addition, the main drawback is the production of autoantibodies, with their related side effects, as mentioned before.

**Figure 3:**
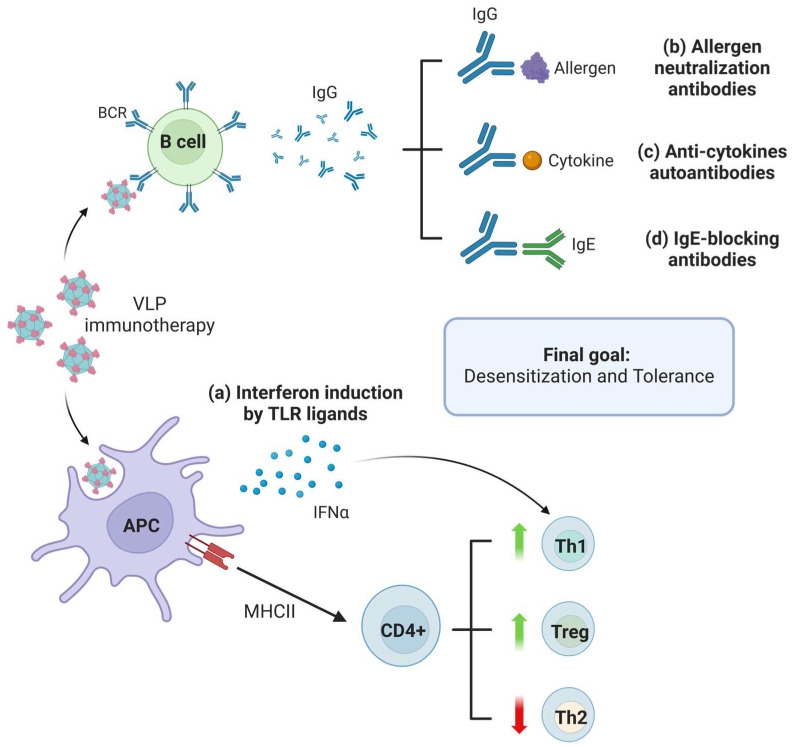
Proposed immune mechanisms associated with the main strategies of VLP immunotherapies against allergies. The strategies share two common mechanisms: The first is based on the recognition of the formula by the BCR of B cells and consequent antibody production. The second consists of the detection of VLPs by APCs and the following presentation to CD4+ lymphocytes, enhancing Th1 and Treg responses with a decrease in the Th2 pathway. Depending on the approach, specific mechanisms will occur: (**a**) TLR ligands coupled to VLP platforms will induce the secretion of class I interferons, skewing the response to a Th1-type. (**b**) Allergen-conjugated VLPs can trigger the production of neutralizing antibodies against the allergen itself. (**c**) Cytokines bound to VLPs can lead to the secretion of anti-cytokines autoantibodies. (**d**) VLPs combined with IgEs elicit the synthesis of anti-IgE antibodies to inhibit the function of the main allergy immunoglobulin. The main goal of these immunotherapies is the desensitization of the patient to the specific allergen and the induction of tolerance (created with BioRender.com).

In summary, Figure 3 aims to depict in a single snapshot the variety of immunological mechanisms behind the use of VLPs to treat allergy, based on the data in this review.

## 4. Conclusions

Virus-like particles have emerged as a novel and promising approach for the treatment of allergic diseases. Within the array of nanoparticles used as biotechnological tools, VLPs show advantages that make them a rational choice. Their structure, resembling their viral origin, along with their repetitive patterns and size is very efficient in promoting entry into the lymphatic system, especially in their uptake and presentation by APCs [103,135]. They show a low or non-toxic profile, are biodegradable and, in many cases, resistant to proteases [54,136]. Moreover, their key capability is to pack molecules of interest within their inner cavity, as well as the possibility of presenting antigens coupled chemically or by genetic fusion [38,137].

Regardless of the coupling method, VLPs display the attached proteins in an ordered and repetitive structure, increasing their immunogenicity, their concentration, and avoiding degradation [138,139]. Thanks to recent advances in genetic engineering, the capacity of VLPs to carry and present more antigens will be optimized. Bioinformatic tools will also be crucial for designing chimeric proteins with improved physicochemical characteristics for antigen displaying in VLPs [140,141]. In this regard, the size of the attached molecule is a very important aspect to consider and may present a challenge for the correct folding of the formula and for antigenic presentation [142,143,144].

Considering that structure and function are closely intertwined, the immunogenic properties must be taken into consideration when optimizing VLPs’ structure. As previously mentioned, the repetitive antigen display in VLPs enhances uptake by APCs, promotes antigen-specific responses, B-cell activation, etc. [52,53,55]. Previous reports have suggested the development of neutralizing IgG/IgM antibodies, which seem to opsonize VLPs, with interesting consequences in their efficacy [145,146]. This type of response needs to be considered when choosing a VLP platform. For example, the viruses on which some VLPs are based, such as norovirus or hepatitis virus, might have been previously presented to our immune system, thus altering the expected response. However, other VLPs, such as plant viruses or bacteriophage Qβ, are immunologically inert [135].

As shown in this review, VLPs are powerful and promising tools, with many in vivo studies, but very few cases reaching clinical trials. This is, in a way, reasonable, given the short period of time since the beginning of using VLPs and the many aspects to be analyzed and optimized. In this regard, it is also important to consider the route of administration, use of adjuvants, quantity, duration, side effects, etc. For instance, there are VLPs presented here that display one allergen on their surface and other examples of VLPs that combine antigen + adjuvant (see Table 1). There are examples of promising results with different presentations, such as those based on VLPs packaging an allergen, where the authors show the establishment of a Th1-Treg response that promotes the reduction of airway hyperresponsiveness [103,147].

These conclusions reflect on the potential of applying VLPs for the treatment of allergic diseases. The data obtained so far show promising results, indicating beneficial immunomodulatory effects and increased safety. Still, there are many unresolved questions to fully understand the mechanisms of protection that VLPs promise, with the final goal of their applicability in human therapy.

## Abbreviations

**AIT**: allergen-specific immunotherapy, **APC**: antigen-presenting cell, **B/IC**: baculovirus/ insect cell, **BCR**: B-cell antigen receptor, **CuMV**: cucumber mosaic virus, **CuMV_TT_**: CuMV with tetanus toxin-derived universal T-cell epitope tt830-843, **cVLP**: chimeric VLP, **DC**: dendritic cell, **EMA**: European Medicines Agency, **eVLP**: enveloped VLP, **FcεRI**: high-affinity receptor for the Fc region of IgE, **FcγRIIb**: Fc fragment of IgG receptor IIb, **FDA**: Food and Drug Administration, **FDC**: follicular dendritic cell, **GMP**: good manufacturing procedures, **HBcAg**: hepatitis B core antigen, **HBV**: hepatitis B virus, **MHC**: major histocompatibility complex, **MLV**: Monoley murine leukemia Virus, **non-eVLP**: non-enveloped VLP, **ODN**: oligodeoxynucleotide, **pDC**: plasmacytoid dendritic cell, **PRR**: pattern recognition receptor, **PTM**: post-translational modification, **TLR**: toll-like receptor, **TuMV**: turnip mosaic virus, **VLP**: virus-like particle.
